# Physical and Dielectric Properties of Ni-Doped In_2_S_3_ Powders for Optical Windows in Thin Film Solar Cells

**DOI:** 10.3390/ma14195779

**Published:** 2021-10-03

**Authors:** Abdelmajid Timoumi, Walid Belhadj, Salah Noaiman Alamri, Mohamed Khalil Al-Turkestani

**Affiliations:** 1 Physics Department, Faculty of Applied Science, Umm AL-Qura University, Makkah P.O. Box 715, Saudi Arabia; wbbelhadj@uqu.edu.sa (W.B.); mkturkestani@uqu.edu.sa (M.K.A.-T.); 2 Physics Department, Faculty of Science, Taibah University, Madinah P.O. Box 30002, Saudi Arabia; alamrisaleh@yahoo.com

**Keywords:** dielectric properties, doping, impedance spectroscopy, In_2_S_3_, nickel, solar cells

## Abstract

This paper reports the effect of Nickel (Ni) on indium sulfide (In_2_S_3_) powder. This work presents a systematic study of the physical and dielectric properties of In_2-x_S_3_Ni_x_ powders with 0, 2, 4, and 6 at.% of nickel. Doped and undoped samples were investigated by X-ray powder diffraction (XRD), energy dispersive X-ray spectroscopy, thermal gravimetric analysis, Fourier transform infrared (FTIR) spectroscopy, Raman spectroscopy, scanning electron microscopy (SEM), and impedance spectroscopy. XRD patterns revealed that each In_2-x_S_3_Ni_x_ composition was crystalline, which was also confirmed by the FTIR results. The presence of Ni in the samples was confirmed by energy dispersive spectroscopy (EDS). The Raman studies show different peaks related to the In_2_S_3_ phase and do not reveal any secondary phases of In–Ni and Ni–S. The SEM images of the undoped and Ni-doped In_2_S_3_ samples indicated a correlation between dopant content and the surface roughness and porosity of the samples. The impedance analysis indicated semiconductor behavior present in all samples, as well as a decrease in resistance with increasing Ni content. This work opens up the possibility of tailoring the properties and integrating Ni-doped In_2_S_3_ nanocomposites as thin film layers in future solar cells.

## 1. Introduction

The inorganic compound indium sulfide (In_2_S_3_) recently gained considerable attention as an optical window material in solar photovoltaic cells. In_2_S_3_ is as well non-toxic and more environmentally friendly compared to other optical window materials such as cadmium sulfide (CdS). In addition, In_2_S_3_ is an n-type semiconductor that crystallizes at atmospheric pressure in three polymorphic forms (named α, β, and γ) as a function of temperature [1]. These remarkable characteristics of In_2_S_3_ combined with its eco-friendly nature [2,3] make it well-suited for use in a variety of applications that are fabricated by different processing methods [4,5,6,7]. In_2_S_3_ is generally used in photovoltaic applications [8], semiconductor batteries [9], and photoelectrochemical cells [10,11]. Our previous research [12,13,14,15,16,17,18,19,20] included detailed investigations of several materials, including thin film indium sulfide. The latter is synthesized in our laboratory and then deposited in the form of thin layers by various techniques such as vacuum thermal evaporation and pyrolysis spray. Various theoretical and experimental studies are made depending on the effect of annealing, temperature, and doping.

In fact, some of the synthesis methods used in our research are relatively complex to manipulate, and a few are costly and time-consuming. Besides our own studies, many other researchers are interested in investigating In_2_S_3_ powders [21,22]. Hamici M. et al. [21] studied the oxidation process of the crystalline powder of In_2_S_3_ and thin films obtained by the Dr. Blade Method. They obtained samples showing a band gap varying continuously between 1.94 eV and 3.72 eV and films of intermediate composition. Gorai S. et al. [22] synthesized the In_2_S_3_ powder and studied its optical characterization and estimated a band gap value around ~2.12 eV. Other studies [23] are also carried out on CdS powder, it having physical properties close to that of In_2_S_3_ and which can be used as a thin film for solar cells.

Recently, 3D transition metal impurities in semiconductor nanoparticles attracted significant interest because of their effect on tuning the emission bands [24,25]. A study of commercially doped In_2_S_3_ will provide an alternative approach for understanding the energy-related applications of this material. In addition, commercial In_2_S_3_ powder is inexpensive and provides similar encouraging results as laboratory synthesized powders.

In this research, we investigated the effects of doping not only on the structural, morphological, and thermal properties, but also on the dielectric properties of Ni-doped and undoped In_2_S_3_ powders. The obtained results are presented and discussed below. To our knowledge, this is the first work on In_2_S_3_ powder doped with nickel.

## 2. Materials and Methods

### 2.1. Materials

In_2_S_3_, a red powder with 99.99% purity, was supplied by the Sigma-Aldrich company and used without purification. Nickel powder (Pulver, Atom Gew. 58.71) in concentrations of 2, 4, and 6 at.% was used to dope In_2_S_3_. The choice of these concentrations is estimated from our previous work, and, generally, there is a limit to the amount of dopant that could be applied. Each sample consisted of 0.2 g of In_2_S_3_. To characterize the electrical properties, the total quantity of each sample was pressed into a pellet 13 mm in diameter and 1 mm thick. Two silver paste electrodes were placed on both sides of the pellet and were connected to copper wires.

### 2.2. Experimental Methods

**Figure 1:**
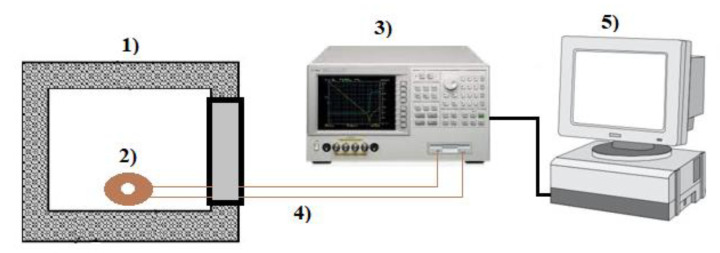
Schematic diagram of experimental system: (**1**) furnace, (**2**) pellet with electrodes, (**3**) impedance analyzer, (**4**) copper wires, and (**5**) computer.

To characterize the structural properties of the In_2-x_S_3_Ni_x_ powder, we used X-Ray diffraction (XRD; Cu-Kα = 1.5418 Å, Shimadzu XRD-6000, Shimadzu, Kyoto, Japan). A thermogravimetric analysis (TGA) was performed on the undoped sample employing a TGA-1000 analyzer (Navas Instruments, Conway, SC, USA). Scanning Electron microscopy (SEM; Supers can SSX-550, Shimadzu, Japan) was used to analyze the surface morphology of the samples. To investigate the crystal phases, we used Raman spectroscopy (RS; 532 nm laser, Senterra, Bruker, Billerica, MA, USA). The complex impedance parameters were investigated as a function of frequency (10 Hz–13 MHz) using a computer-controlled impedance analyzer (Agilent 4294A, Agilent, Santa Clara, CA, USA). The entire experimental system is shown in Figure 1.

## 3. Results and Discussion

### 3.1. XRD and EDS Analysis

**Figure 2:**
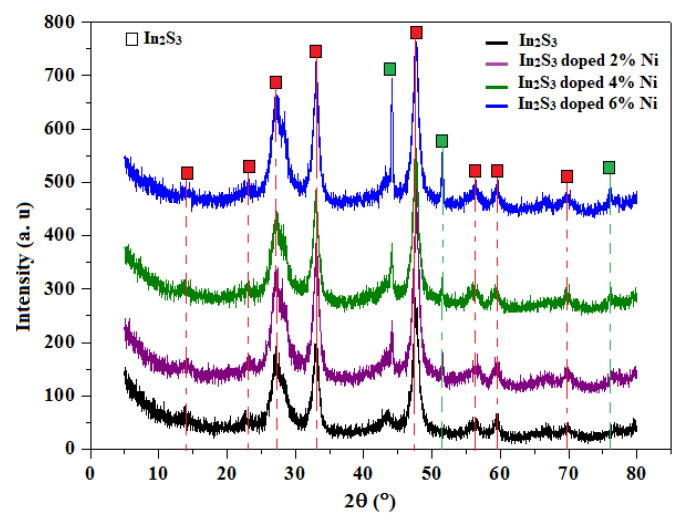
XRD patterns of In_2_S_3_ and In_2_S_3_ doped with (2, 4, and 6 at.%) Ni.

X-ray diffraction (Cu–Kα = 1.5418 Å) was used to analyze the structures of the In_2_S_3_ and In_2-x_S_3_Ni_x_ samples. Figure 2 shows that all samples were crystalline. This result agrees with that obtained by Lucena et al. [26]. The appearance of other peaks is also apparent depending on the increase in the dopant level at 2θ angles of 44°, 52°, and 77°, as indicated by the green squares in Figure 2. These peaks are most likely the residual stress in the sample caused by the difference in ionic size between Ni^2+^ (0.70 Å) and In^3+^ (0.80 Å) [27]. No other phase corresponding to the nickel impurity was detected, which is explained by the fact that the Ni ions occupy sulfur substitution positions and do not change the crystal system of the material.

**Figure 3:**
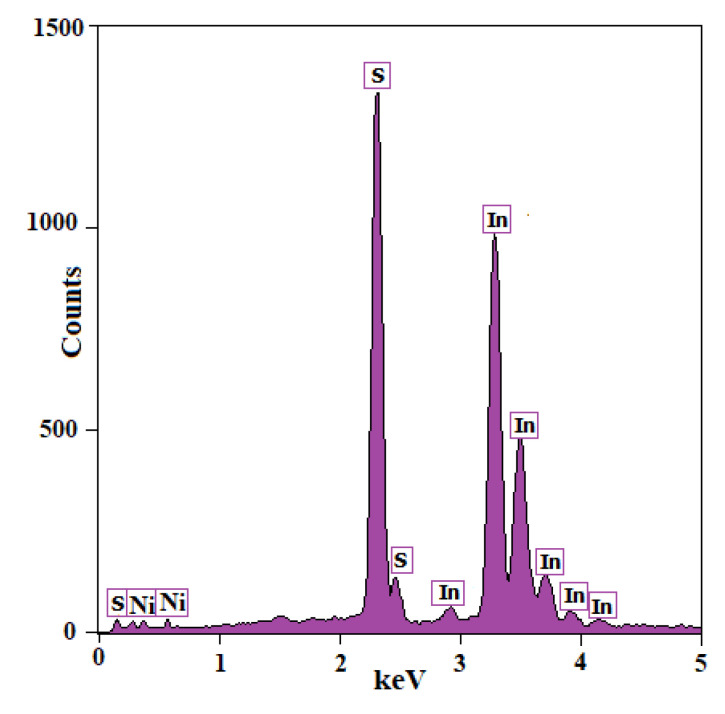
The EDS pattern of the undoped In_2_S_3_ powder.

A compositional analysis was performed using energy-dispersive X-ray spectroscopy (EDS). The EDS pattern of the undoped In_2_S_3_ samples (Figure 3) shows the presence of indium and sulfur elements in an atomic percentage ratio of 2/3 for In:S. The other unidentified peaks were attributed to the glass substrate.

The EDS analysis also confirmed the presence of different nickel contents for each sample. The atomic percentages of the elements in the samples are listed in Table 1, which indicates that when the Ni content increased, the quantity of indium decreased slightly, and the percentage of sulfur remained constant. It is expected that Ni doping of the In_2_S_3_ samples was substitutional.

### 3.2. Thermal Analysis

**Figure 4:**
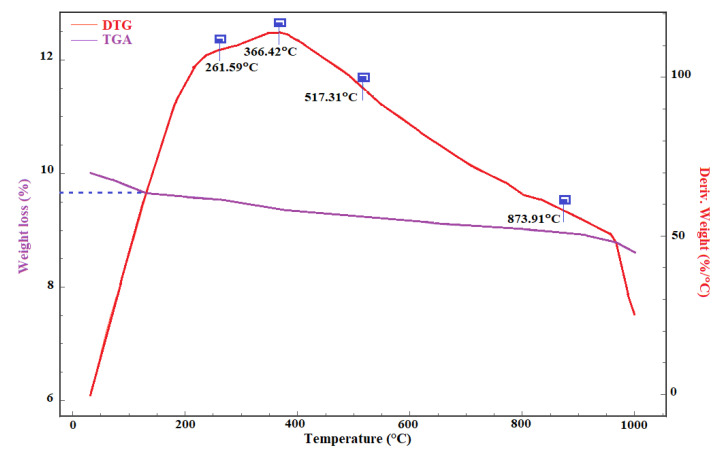
TGA and DTG analysis of the undoped In_2_S_3_ powder.

The thermal behavior of the In_2_S_3_ material was studied using a TGA. As shown in Figure 4, the TGA thermograms show that the untreated In_2_S_3_ material is thermally stable at temperatures beyond 400 °C, and the weight loss is approximately 5%. This behavior is attributed to the purity and crystallinity of the material.

### 3.3. Raman Analysis

**Figure 5:**
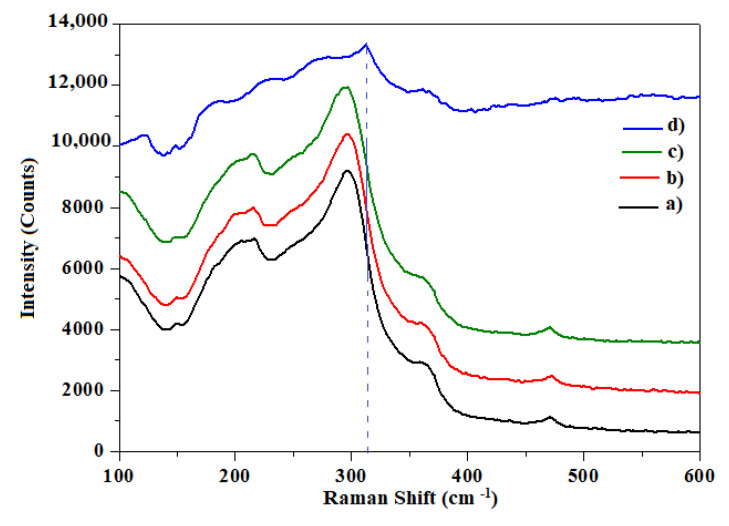
Raman spectra of (**a**) undoped In_2_S_3_ and In_2_S_3_ doped with (**b**) 2 at.%, (**c**) 4 at.%, and (**d**) 6 at.% Ni.

Figure 5 presents the experimental Raman spectra for all samples in the range 100–600 cm^−1^, which indicates the presence of In_2_S_3_ phase modes, in agreement with the results obtained by Kraini et al. [28]. The active modes were observed at 115, 210, 308, and 365 cm^−1^, confirming their composition and structure [29]. The indicated modes produced broad peaks with low intensity. At about 220 cm^−1^, the observed peak corresponds to the presence of elemental sulfur in the structure of the thin film. At near 300 cm^−1^, the obtained Raman mode is attributed to the Eg mode of the In–S bond. The position of the peak and the half-width of the phase of Raman bands were independent of the Ni-doping content. However, there was a slight change in the relative intensity of the Raman band at 315 cm^−1^, which appears to correlate with the changes observed by X-ray diffraction for the sample containing 6 at.% Ni. We also noticed the absence of secondary phases, which is in good agreement with the obtained XRD results.

### 3.4. FTIR

**Figure 6:**
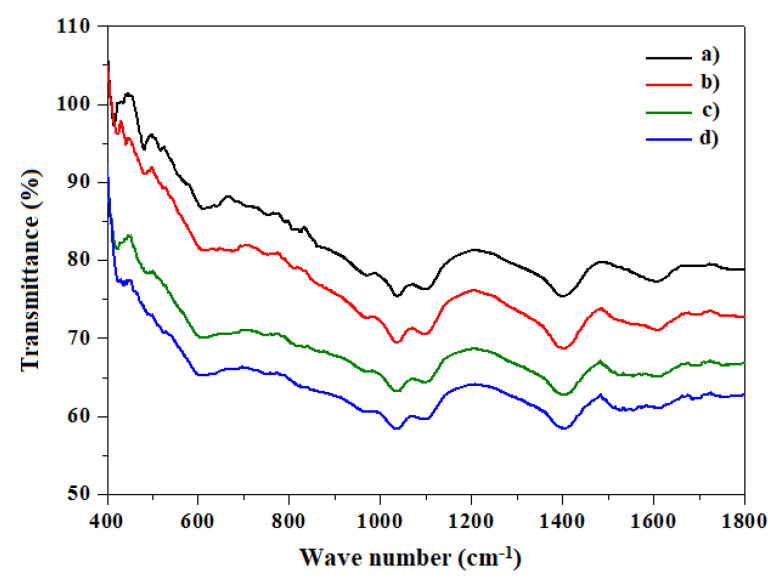
FTIR spectra of (**a**) undoped In_2_S_3_ and In_2_S_3_ doped with (**b**) 2, (**c**) 4, and (**d**) 6 at.% Ni.

FTIR spectral fingerprints for each sample in the wavenumber range of 100 to 600 cm^−1^ provided details of the chemical structure. Figure 6 shows the presence of three absorption bands at 1000–1500 cm^−1^ corresponding to the extension of the C–H and O–H bonds, and also at 900–1200 cm^−1^ and approximately 800 cm^−1^ corresponding to the stretching of the C=O bonds and the O–H band, respectively.

### 3.5. SEM

**Figure 7:**
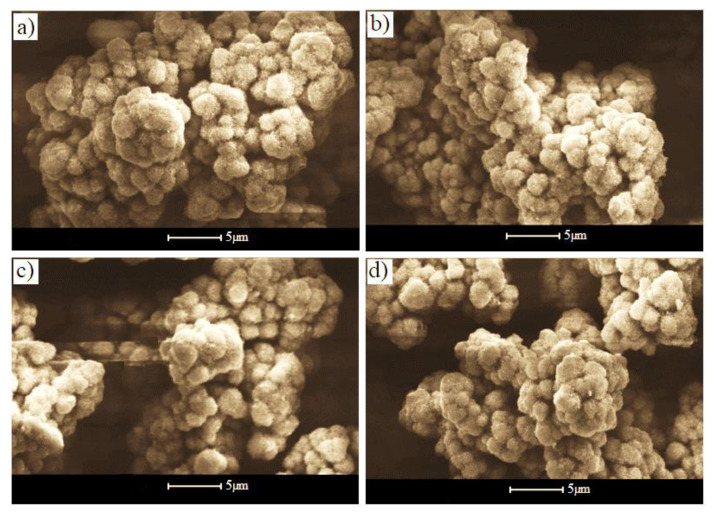
SEM images of (**a**) undoped In_2_S_3_ and In_2_S_3_ doped with (**b**) 2, (**c**) 4, and (**d**) 6 at.% Ni.

Figure 7 shows the SEM images of the undoped In_2_S_3_ and Ni-doped In_2_S_3_ samples. The surfaces show highly irregular surface roughness and porosity that increase with increasing the Ni-doping concentration, as shown in Figure 7d. There was also a clear increase in the number of grains with increasing Ni content, as well as surface homogeneity. This is probably due to Ni filling the pores on the surface of the composite layer. From the SEM images, the surface roughness obviously increased after doping.

### 3.6. Electrical Impedance

**Figure 8:**
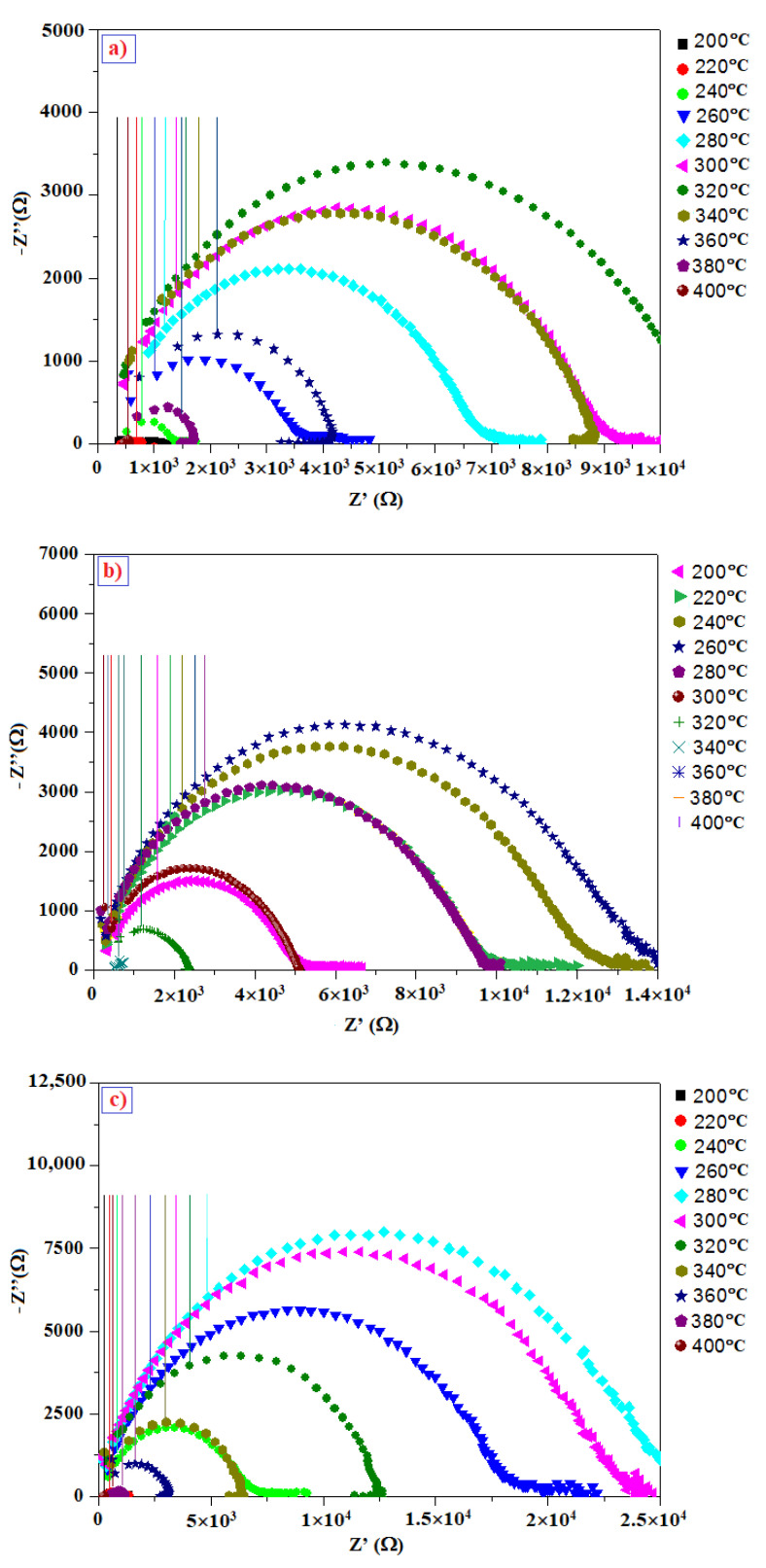

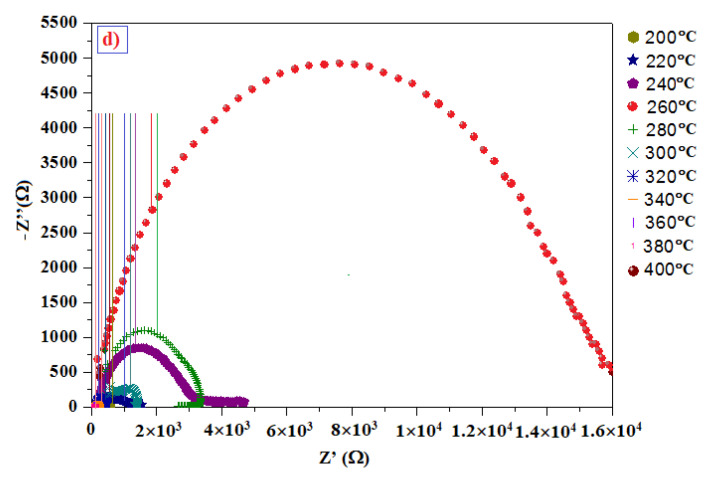
(-Z′′ vs. Z′) plots of (**a**) In_2_S_3_ and In_2_S_3_ doped with (**b**) 2, (**c**) 4, and (**d**) 6 at.% Ni and heated to 200–400 °C.

Currently, much work is interested in electrical and dielectric properties of materials [30,31,32]. Nyquist plots are commonly used for characterizing electrical and dielectric properties of semiconductors’ materials [33,34,35,36], so we constructed Nyquist plots for our In_2_S_3_ powder samples. Doping is one of the approaches used to minimize electrical resistivity [37]; therefore, to study the dopant effects on the dielectric behavior of the In_2_S_3_ samples, we used impedance spectroscopy (IS). The undoped and doped In_2_S_3_ powders were pressed into a pellet (13 mm diameter and 1 mm thickness) prior to applying silver paste, which served as an electrical contact. The Nyquist plots obtained for all samples in the frequency range of 10 Hz to 30 MHz and at different temperatures of 200–400 °C are presented in Figure 8. These Nyquist plots exhibit semi-circular arcs whose centers are located under the real part axis, which suggests a non-Debye-type relaxation phenomenon of the charge carriers [38]. The diameter and shape of the semicircles changed with the temperature, which indicates temperature-dependent semiconductor behavior [39,40]. Initially, the Nyquist diagrams illustrate increases in the semi-circular diameters as the temperature increases above 200 °C [33,41]. However, the semi-circles shifted to lower frequencies, and their diameters decreased after heating to 260–320 °C, indicating a thermally activated electrical conductivity and relaxation time distribution [42]. This phenomenon was observed at 320 °C for pure In_2_S_3_ and from 280 °C to 260 °C for the Ni-doped samples. Resistance values decreased rapidly with temperature, and the carrier concentration increased with doping because indium sulfide has many vacant indium sites [43].

**Figure 9:**
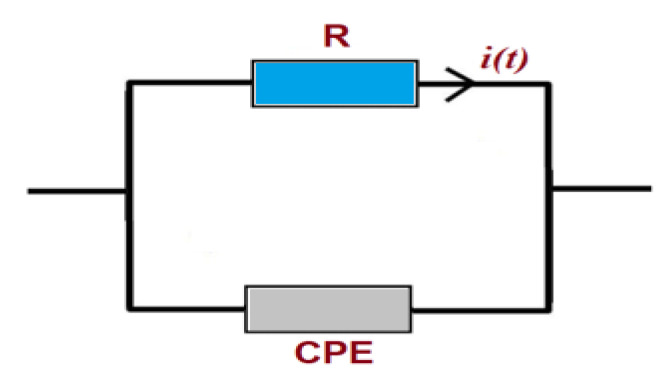
The proposed equivalent circuit.

The modeling of the measured impedance spectra and the choice of an appropriate electrical equivalent circuit allow us to consider the electrical phenomena in our undoped and Ni-doped In_2_S_3_ material. The ZSimpWin 3.10 program was used to compute the circuit parameters. The assigned equivalent circuit for this study is shown in Figure 9, in which the resistance is connected in parallel to the constant phase element impedance (CPE).

The CPE impedance (*Z_CPE_*) is defined in Equation (1) [44];

(1) $$Z_{CPE}=\frac{1}{Q{\left(j\omega \right)}^{\beta }}$$

where:

Q is a constant value independent of frequency; Ω is the angular frequency; and β is an exponent that measures the arc depression of the CPE impedance [45].

## 4. Conclusions

In summary, we investigated the effect of doping In_2_S_3_ powder with nickel on the crystal structure, distribution of dopants, thermal behavior, molecular structure and bonding modes, surface morphology, and electrical impedance. This study showed that Ni doping preserved the crystallinity of each dopant concentration, and no phase changes were observed due to Ni doping. The EDS analysis proved that the In_2_S_3_ samples had a Ni-rich and S-deficient composition. The SEM images presented in this work showed a gradual formation of dense samples that were free of cracks with increasing Ni content. The electrical conductance improved with increasing the temperature from a certain characteristic value of the sample. The results obtained in this study highlight the significant effect of doping on the electrical and structural properties of In_2_S_3_ especially in its powder form. The results reported in this study may facilitate the use of In_2_S_3_ materials for future applications in photovoltaic devices.

## Figures and Tables

**Table 1:** Chemical composition of In_2_S_3_ and In_2_S_3_:Ni samples.

Ni:In Ratio in Powder Samples	Elements		
	In (at.%)	S (at.%)	Ni (at.%)
**Undoped**	40.54	59.46	0
**2%**	39.47	58.50	2.04
**4%**	40.12	57.08	2.80
**6%**	39.63	56.70	3.67

## Data Availability

The data presented in this study are available on request from the corresponding author.
